# *Toxoplasma gondii* dense granule protein 3 promotes endoplasmic reticulum stress-induced apoptosis by activating the PERK pathway

**DOI:** 10.1186/s13071-022-05394-5

**Published:** 2022-08-02

**Authors:** Cudjoe Obed, Minmin Wu, Ying Chen, Ran An, Haijian Cai, Qingli Luo, Li Yu, Jie Wang, Fang Liu, Jilong Shen, Jian Du

**Affiliations:** 1 Department of Biochemistry and Molecular Biology, School of Basic Medical Sciences, Anhui Medical University, Hefei, 230032 China; 2 The Research Center for Infectious Diseases, School of Basic Medical Sciences, Anhui Medical University, Hefei, 230032 China; 3 The Provincial Key Laboratory of Zoonoses of High Institutions in Anhui, Anhui Medical University, Hefei, 230032 China; 4 The Key Laboratory of Microbiology and Parasitology of Anhui Province, Anhui Medical University, Hefei, 230032 China; 5 School of Nursing, Anhui Medical University, Hefei, 230032 China; 6 Department of Microbiology & Immunology School of Medical Sciences, University of Cape Coast, Cape Coast, Ghana

**Keywords:** *Toxoplasma gondii*, GRA3, Endoplasmic reticulum, ER stress, UPR, Apoptosis

## Abstract

**Background:**

*Toxoplasma gondii* is a neurotropic single-celled parasite that can infect mammals, including humans. Central nervous system infection with *T. gondii* infection can lead to *Toxoplasma* encephalitis. *Toxoplasma* infection can cause endoplasmic reticulum (ER) stress and unfolded protein response (UPR) activation, which ultimately can lead to apoptosis of host cells. The dense granule protein GRA3 has been identified as one of the secretory proteins that contribute to the virulence of *T. gondii*; however, the mechanism remains enigmatic.

**Methods:**

The expression of the GRA3 gene in RH, ME49, Wh3, and Wh6 strains was determined using quantitative real-time polymerase chain reaction (qRT–PCR). pEGFP-GRA3_Wh6_ was constructed by inserting Chinese 1 Wh6 GRA3 (GRA3_Wh6_) cDNA into a plasmid encoding the enhanced GFP. Mouse neuro2a (N2a) cells were transfected with either pEGFP or pEGFP-GRA3_Wh6_ (GRA3_Wh6_) and incubated for 24–36 h. N2a cell apoptosis and ER stress-associated proteins were determined using flow cytometry and immunoblotting. Furthermore, N2a cells were pretreated with GSK2656157 (a PERK inhibitor) and Z-ATAD-FMK (a caspase-12 inhibitor) before GRA3_Wh6_ transfection, and the effect of the inhibitors on GRA3_Wh6_-induced ER stress and apoptosis were investigated.

**Results:**

GRA3 gene expression was higher in the less virulent strains of type II ME49 and type Chinese 1 Wh6 strains compared with the virulent strains of type I RH strain and type Chinese 1 Wh3 strain. Transfection with GRA3_Wh6_ plasmid induced neuronal apoptosis and increased the expression of GRP78, p-PERK, cleaved caspase-12, cleaved caspase-3, and CHOP compared with the control vector. Pretreatment with GSK2656157 and Z-ATAD-FMK decreased apoptosis in N2a cells, and similarly, ER stress- and apoptosis-associated protein levels were significantly decreased.

**Conclusion:**

GRA3 induces neural cell apoptosis via the ER stress signaling pathway, which could play a role in toxoplasmic encephalitis.

**Graphical Abstract:**

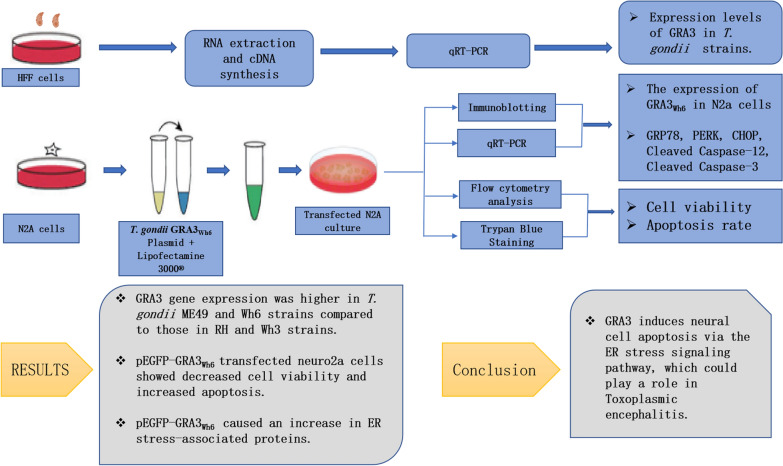

## Background

*Toxoplasma gondii* is a common intracellular coccidian parasite that infects human beings and animals [1–3]. Most *T. gondii* infections are usually asymptomatic and result in a self-limiting disease in immunocompetent hosts [4]; however, in chronic immunocompromised individuals, in particular patients with HIV, *T. gondii* can cause severe and fatal tissue damage [4, 5]. In pregnant women, *T. gondii* can cause miscarriage or deleterious effects to infants or newborns [6–8]. Due to its preference for neural cells, *Toxoplasma* infection is responsible for neurological manifestations, including encephalitis, intracranial calcifications, and hydrocephalus [9–11].

Several conditions, including infectious and neurodegenerative diseases, are known to cause a build-up of misfolded proteins within the endoplasmic reticulum that interfere with the normal functioning of the endoplasmic reticulum (ER). This leads to ER stress [12, 13]. To alleviate the effect of the stress, ER-localized transmembrane signal proteins activate the unfolded protein response (UPR) to restore protein homeostasis [14, 15]. However, an unremitting UPR can activate UPR-mediated inflammatory and apoptotic pathways, resulting in cell death [16, 17].

Previous studies have demonstrated that the Chinese 1 Wh3 and type I RH strains can induce neural stem cell apoptosis via the ER stress-mediated apoptosis signaling pathway [18, 19]. Additionally, secretory proteins such as ROP18 and GRA15 have been demonstrated to induce apoptosis of neural cells [10, 19] and carcinoma JEG-3 cells [20], respectively. In our recent study, we demonstrated that *Toxoplasma* ROP18 phosphorylates the host RTN1-C to trigger ER stress-mediated neural apoptosis by enhancing glucose-regulated protein 78 (GRP78) acetylation [11].

Among the extensively studied dense granule proteins (GRAs) in *T. gondii*, GRA3 is believed to interact with the host cell endoplasmic reticulum (ER) via calcium modulating cyclophilin ligand (CAMLG) [21, 22]. GRA3 is a 29 kDa dense granule protein localized to the parasitophorous vacuole membrane and intravacuolar network. Aside from its physiological role in the uptake of nutrients from host cells, GRA3 in type II strains has also been identified to contribute to its virulence [21, 23]. Herein, we demonstrate for the first time that *Toxoplasma* GRA3 induces apoptosis in infected neuro2a (N2a) cells by activating the protein kinase R (PKR)-like ER kinase (PERK) pathway to initiate the apoptotic cascade.

## Methods

### Parasite and cell culture

*Toxoplasma gondii* RH, ME49, Wh6, and Wh3 tachyzoites were cultured in human foreskin fibroblast (HFF) cells in Dulbecco’s modified Eagle’s medium (DMEM) which contained 10% fetal bovine serum (FBS) and 1% penicillin–streptomycin amphotericin B (Biological Industries, Israel). N2a cells were cultured and maintained in DMEM which contained 10% FBS and 1% penicillin–streptomycin–amphotericin B (Biological Industries, Israel) at 37 °C in a 5% CO_2_ humidified atmosphere. Cells were serially passaged when they reached 80–90% confluence. HFF and N2a cells were regularly inspected for mycoplasma contamination.

### Extraction of RNA and cDNA synthesis

Total RNA from *T. gondii* tachyzoites (RH, ME49, Wh6, and Wh3 strains) was obtained using TRIzol reagent (Invitrogen, CA, USA) following the manufacturer’s protocols. Extracts with A260/A280 and A260/A230 absorbance ratios between 1.92 and 2.20 were considered pure and free from contaminating reverse transcriptase or DNA polymerase inhibitors and were examined on 1% agarose gels. The concentration of purified RNA was measured using a NanoDrop™ One (Thermo Scientific, CA, USA). RNA extracts were stored in a −80 °C refrigerator for subsequent use. Complementary DNA (cDNA) was synthesized from total RNA samples to using a RevertAid First Strand cDNA Synthesis Kit (Thermo Fisher, USA) following the manufacturer’s recommendations. A 1:10 dilution of the cDNA reaction was prepared, measured, and stored at −20 °C for use in subsequent steps.

### Plasmid construction

The open reading frame (*ORF*) encoding *T. gondii* GRA3 (http://toxodb.org) cDNA was amplified from Wh6 tachyzoite RNA using real-time (RT)-PCR. The oligonucleotides used included ME49-GRA3-EcorI-GFP (5ʹ-CGGAATTCATGGACCGTACCATATG 3ʹ; the EcoRI site is underlined) and the reverse primer ME49-GRA3-SalI-GFP (5ʹ-GTCGACTTATTTCTTGGAGGCTTTG 3ʹ; the Sal1 site is underlined). GRA3 primer synthesis and gene sequencing were performed by General Biosystems Co., Ltd. (Anhui, China). A pEGFP-C2 vector (BD Biosciences Franklin Lakes, NJ, USA) was used to construct the pEGFP-GRA3_Wh6_ plasmid by inserting digested *Tg*GRA3_Wh6_ cDNA into the digested pEGFP-C2 vector. The resulting pEGFP-GRA3_Wh6_ plasmid was transformed into *Escherichia coli* TOP10 (Invitrogen Corp., USA) and screened.

### Transfection of N2a cells with pEGFP and pEGFP-GRA3_Wh6_ cDNA

Plasmid transfection was performed using Lipofectamine 3000 transfection reagent (Thermo Fisher Scientific, China) in 6-well plates following the manufacturer’s instructions. In brief, cells were seeded at a density of 1 × 10^5^ cells/ml in 6-well plates and cultured to reach between 70 and 90% confluence at the time of transfection. DNA plasmid (2.5 µg) was diluted in 125 µl Opti-MEM containing 5 µl P3000 reagent, and 5 µl Lipofectamine 3000 transfection reagent was diluted in 125 µl Opti-MEM. The diluted Lipofectamine 3000 transfection reagent and diluted DNA were mixed gently and incubated at room temperature for 15 min. Following incubation, a total of 250 µl DNA-lipid complex was gently pipetted into each well. Control wells contained only cultured N2a medium. Cells were incubated between 18 and 48 h, and the expression levels of the pEGFP vector and pEGFP-GRA3_Wh6_ (GRA3_Wh6_) were visualized using an Olympus IX51 fluorescence microscope (Japan). After transfection, the cells were treated and analyzed by immunoblotting unless otherwise indicated.

### Induction of ER stress

ER stress and apoptosis in N2a cells were induced using 4 µg/ml tunicamycin (TM) and 0.5 µM staurosporine (STS), respectively (MedChemExpress LLC, Shanghai, China), following the manufacturer’s protocols.

### Treatment with inhibitors

After seeding N2a cells for 24 h, cells were pretreated with 4 µM GSK2656157 (MedChemExpress LLC, Shanghai, China) (a PERK inhibitor) and 5 µM Z-ATAD-FMK (BioVision Inc., Milpitas, CA, USA) (a caspase-12 inhibitor) for 1.5 h and 6 h, respectively. Following pretreatment with inhibitors, Lipofectamine 3000 reagent was used to transfect cells with plasmids as described previously.

### Apoptosis detection

Apoptosis of N2a cells was determined using the PE-Annexin V/7-AAD (BD Biosciences, USA) staining method following the manufacturer’s instructions. In brief, cells in each well were washed twice with cold phosphate-buffered saline (PBS) and harvested using 0.25% trypsin solution. Growth medium was added to inactivate trypsin. Supernatants from each well were transferred to Eppendorf tubes and centrifuged at 800 RPM for 3 min. Pellets were then resuspended in 100 µl 1× Annexin V binding buffer. Five microliters of PE-Annexin V and 5 µl of 7-AAD were added to the cell suspensions and mixed gently. Cells were incubated in the dark at room temperature for 15 min. After incubation, 300 µl of Annexin V binding buffer was added to each test tube. Apoptosis of N2a cells was detected using a FACSCalibur flow cytometer (BD Biosciences, USA) within 1 h, and the data were analyzed using FlowJo/CytExpert software. Annexin V+/7-AAD represented cells in the early-stage apoptosis, while Annexin V+/7-AAD+ represented cells in the late-stage apoptosis. Mock-transfected cells represented negative control, whereas STS- and TM-treated cells served as positive controls.

### Cell viability assay

Cell viability was measured using the Trypan Blue Staining Cell Viability Assay Kit (Beyotime, Shanghai, China). Briefly, cells were seeded at a density of 1 × 10^5^ cells/ml in 6-well plates. After transfection and/or pretreatment with inhibitors, N2a cells were collected and stained with trypan blue solution for 3 min at room temperature. The stained cells and total number of cells were counted using a hemocytometer. The cell viability was calculated using the equation:

$$ {\text{Cell viability}}\, = \,\frac{{\left( {{\text{total number of cells}} - {\text{number of stained cells}}} \right)}}{{{\text{total number of cells}}\, \times \,{1}00\% }} $$

### Immunoblotting

N2a cells were harvested 24 h after plasmid transfection, and the expression levels of GRA3, phospho-PERK (p-PERK), glucose-regulated protein (GRP)-78, cleaved caspase-12, cleaved caspase-3, and C/EBP-homologous protein (CHOP) were determined by immunoblotting. In brief, cells were washed with cold PBS and lysed in RIPA lysis buffer (Beyotime Institute of Biotechnology, Jiangsu, China). The cell lysate was centrifuged at 16,000×*g* for 10 min at 4 °C, and the supernatant was collected. Proteins (40 μg) were separated on 10–12% sodium dodecyl sulfate–polyacrylamide gel electrophoresis (SDS–PAGE) gels and transferred onto 0.45 µM nitrocellulose membranes (Millipore, Billerica, MA, USA). The blotting membranes were then blocked with 5% skimmed milk in 1× TBST for an hour and incubated with primary antibodies (1:1000 dilution) overnight at 4 °C. Blots were subsequently incubated for 1 h with the respective secondary antibodies (1:4000 dilution) at room temperature. Blots were washed and probed with an ECL kit (Affinity Bioscience Ltd., Jiangsu, China). Images from blots were viewed using a Bio-Rad ChemiDoc XRS+ imaging system, and ImageJ software (Rawak Software, Inc., Stuttgart, Germany) was used to calculate the relative optical densities of each band. The relative protein expression levels were normalized to that of β-actin. Mock-transfected cells served as a negative control. Rabbit anti-caspase-12, rabbit anti-caspase-3, mouse anti-CHOP, mouse anti-β-actin, goat anti-rabbit immunoglobulin G (IgG), and goat anti-mouse IgM were all purchased from Proteintech (Wuhan, Hubei, China). Rabbit anti-p-PERK was purchased from Affinity Bioscience Ltd. (Jiangsu, China), rabbit anti-PERK was purchased from Cell Signal Technology, Inc. (Danvers, MA, USA), rabbit anti-GRP78 was purchased from Abcam (Cambridge, UK), and rabbit anti-GFP was purchased from Santa Cruz Biotechnology (Dallas, TX, USA).

### Real-time PCR

Total RNA was obtained from the cells by the TRIzol method as described previously. cDNA was synthesized from purified RNA using a RevertAid First Strand cDNA Synthesis Kit (Thermo Fisher Scientific, USA). Reverse transcriptase quantitative polymerase chain reactions (RT-qPCR) were performed using SYBR Green ProTaq (TaKaRa, Tokyo, Japan) following the manufacturer’s protocols. The forward and reverse primers listed in Table 1 were synthesized by General Biosystems Co., Ltd. (Anhui, China). Briefly, 1 µg of RNA was reverse transcribed to a 10 µl final volume master mix reaction. Two microliters of cDNA, 0.4 µl of forward and reverse primers, 5 µl of SYBR Green ProTaq and 2.2 µl of double-distilled (dd)H_2_O were added to achieve a final reaction volume of 10 µl. PCR was carried out for 45 cycles of initial denaturation for 5 s at 94 °C, annealing for 15 s at 55 °C, and extension for 1 min at 72 °C using a LightCycler 96 (Roche, Basel, Switzerland). All RT-qPCR reactions were carried out in triplicate. Gene expression levels were normalized to actin levels, and data were quantified with the delta-delta CT (ΔΔCT) method.

**Table 1:** Oligonucleotide sequences for Mus musculus genes and *Tg*GRA3 (RT-qPCR)

Primer name	Sequence (5ʹ–3ʹ)
Caspase-12-F	ACAAAGGGATAGCCACTGCT
Caspase-12-R	ACCAGTCTTGCCTACCTTCC
Caspase-3-F	AAGGAGCAGCTTTGTGTGTG
Caspase-3-R	GGCAGGCCTGAATGATGAAG
PERK-F	CGGCAGGTCCTTGGTAATCA
PERK-R	CGTCCAAATCCCACTGCTTT
CHOP (C/EBP)-F	TCGCTCTCCAGATTCCAGTC
CHOP (C/EBP)-R	ACTGACCACTCTGTTTCCGT
GRP78-F	GGTGGGCAAACCAAGACATT
GRP78-R	TCAGTCCAGCAATAGTGCCA
Actin-F	AACTAGGCTGCTCCCTGAAG
Actin-R	TGCAAAGGATCCCGCTTAGA
GRA3-F	TTCTCGCCGCCTACTACATT
GRA3-R	TGTGTCCAATCTGCGTCAAC

*PERK* protein kinase R (PKR)-like ER kinase, *GRP78* 78-kDa glucose-regulated protein, *CHOP* C/EBP homologous protein, *GRA3* dense granule proteins

### Statistical analysis

Data are presented as mean ± standard deviation (SD) of three or more independent experiments. A two-tailed independent Student *t*-test was used to compare the differences between two groups. One-way analysis of variance (ANOVA) was used to compare the differences among multiple groups. A *P* < 0.05 was considered statistically significant. Analysis was performed using GraphPad Prism 8 Version 8.02.

## Results

### High expression of GRA3 in type II ME49 and Chinese 1 Wh6 strains

**Fig. 1:**
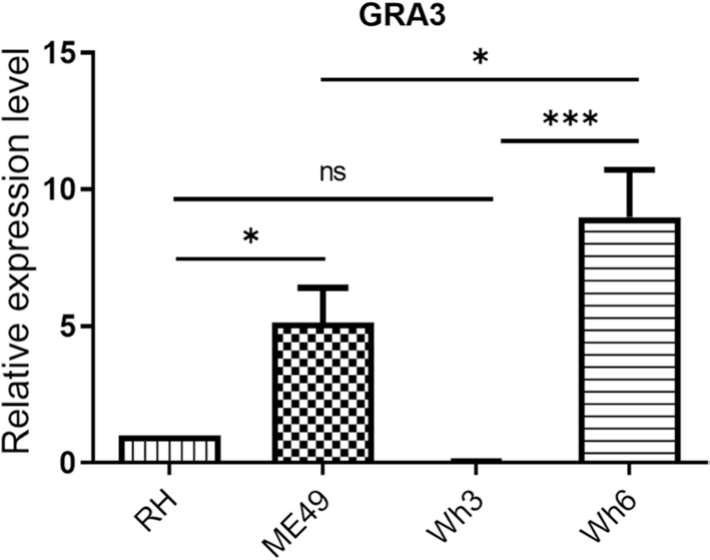
Dense granule protein 3 (GRA3) gene expression among different isolates. GRA3 expression levels were compared between virulent RH and Wh3 strains and less virulent ME49 and Wh6 strains. The RT-qPCR was performed in triplicate and values were expressed as mean ± SD. *P < 0.05, ***P < 0.001, and ns, not statistically significant

To understand the role of GRA3 in the virulence of *T. gondii,* first, GRA3 primers were used to amplify the GRA3 sequence in cDNA obtained from the wild-type (RH), type II strain (ME49), and the Chinese 1 Wh3 (virulent) and Wh6 (less virulent) parasite strains, and GRA3 messenger (mRNA) expression levels were determined in each strain using RT-qPCR. Significant increases in GRA3 expression were observed in the ME49 (*P* < 0.05) and Wh6 (*P* < 0.001) strains compared with the RH and Wh3 strains, respectively. However, the expression level of GRA3 in the Wh6 strain was higher than that in the ME49 strain (*P* < 0.05) (Fig. 1). As such, we sought to compare the DNA sequences between GRA3_Wh6_ and GRA3_ME49_ . The sequence alignment results revealed that GRA3_Wh6_ was as same as GRA3_ME49_, which is consistent with our previous study [24]. Because the highest expression of GRA3 was found in the Wh6 strain compared with RH, ME49, and Wh3 strains, we adopted GRA3_Wh6_ for subsequent experiments.

### Expression of GRA3_Wh6_ in N2a cells

**Fig. 2:**
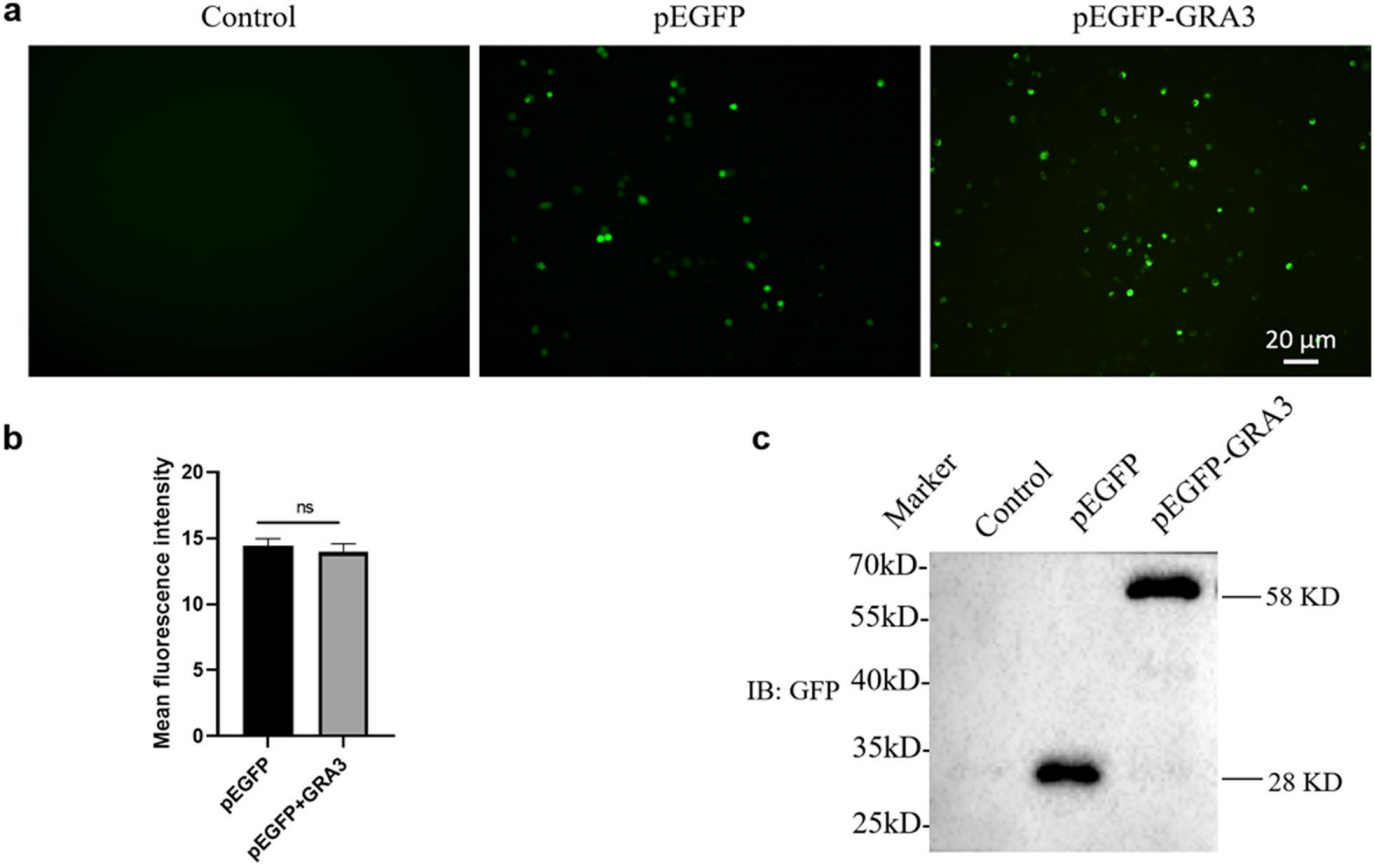
Expression of dense granule protein 3 (GRA3_Wh6_). Mouse neuroblastoma (N2a) cells were transfected with either a control vector (pEGFP, plasmid encoding enhanced green fluorescent protein) or pEGFP-GRA3_Wh6_ fusion protein (GRA3_Wh6_) for a period of 24 h. Mock-transfected N2a cells served as a negative control. **a** The expression of green fluorescent protein (GFP) in pEGFP and pEGFP-GRA3_Wh6_ transfected N2a cells was observed using fluorescence microscopy. Scale-bar 20 µM. **b** The GFP intensity in pEGFP and pEGFP-GRA3_Wh6_-transfected N2a cells was measured using ImageJ. ns, not statistically significant. **c** The expression of GRA3_Wh6_ was confirmed by immunoblotting. Molecular weight (MW) of GFP = 28k Da, MW of pEGFP-GRA3_Wh6_ fusion protein = 58 kDa

To determine whether GRA3_Wh6_ plasmid could be efficiently expressed in neural cells, we transfected pEGFP or pEGFP-GRA3_Wh6_ (GRA3_Wh6_) into the mouse neuroblastoma N2a cells and determined the expression of GRA3_Wh6_ protein. Twenty-four hours after transfection, GFP fluorescence was observed in both pEGFP- and pEGFP-GRA3_Wh6_-transfected N2a cells. The fluorescence signal in pEGFP-GRA3_Wh6_-transfected N2a cells was comparable to that in pEGFP-transfected cells (Fig. 2a, b). pEGFP and pEGFP-GRA3_Wh6_ fusion protein were blotted at 28 kDa and 58 kDa following a 24 h transfection (Fig. 2c). We noted that the observed molecular weight of GRA3 was ~ 30 kDa, which was similar to that of previous studies [21, 23].

### Reduced cell viability and apoptosis of neuronal cells transfected with GRA3_Wh6_ in vitro

**Fig. 3:**
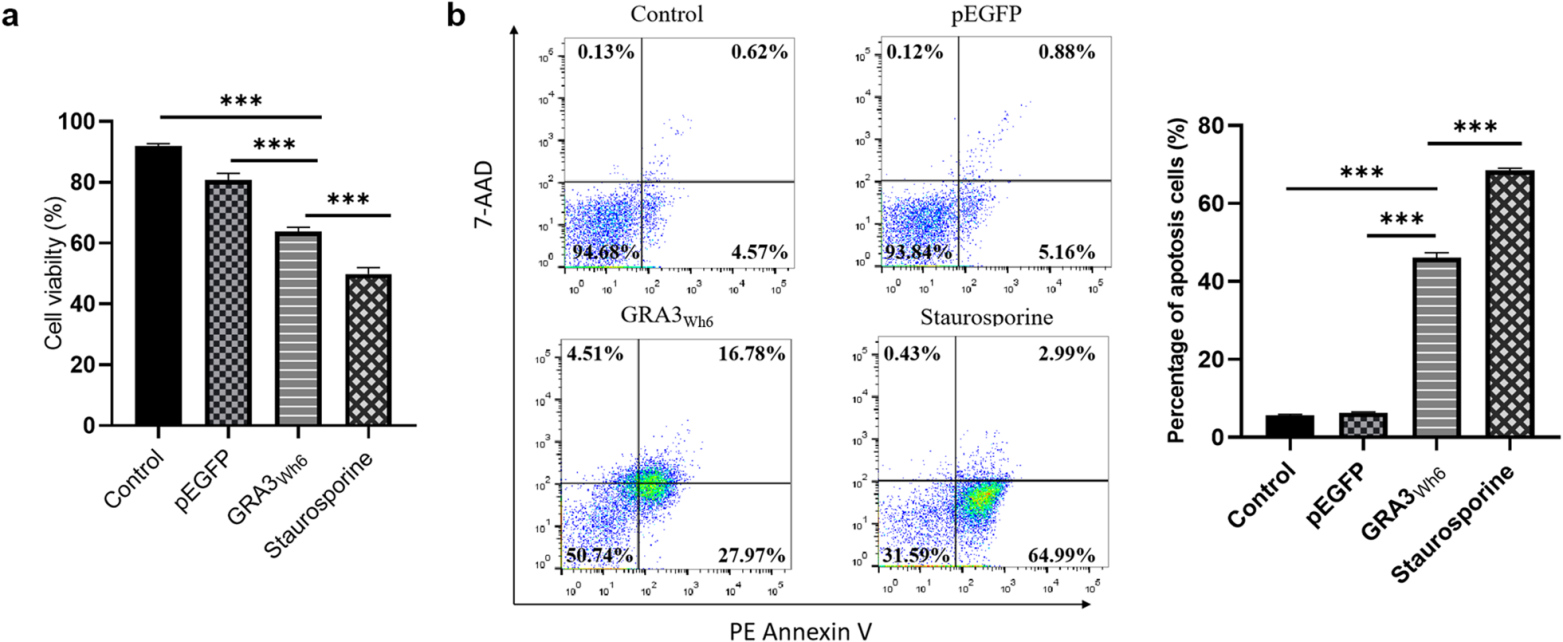
Dense granule protein 3 (GRA3_Wh6_)-induced loss of cell viability and apoptosis. N2a cells were transfected with either a control vector (pEGFP) or pEGFP-GRA3_Wh6_ (GRA3_Wh6_) for a 24 h period. Mock-transfected N2a cells served as the negative control, and N2a cells treated with staurosporine (1 μM, 12 h) served as the positive control. **a** Cell viability was measured using the trypan blue staining cell viability assay. **b** Apoptosis of cells was determined using flow cytometry after staining with Annexin V-PE/7-AAD. The plots are from a representative measurement and the data were expressed as mean ± SD in three different assays (*n* = 3). ***P < 0.001

To investigate whether GRA3 could impact the survival of neural cells, the cell viability and rate of apoptosis in N2a cells transfected with either pEGFP or pEGFP-GRA3_Wh6_ (GRA3_Wh6_) were analyzed. The viability of mock-transfected N2a cells was 91.89% whereas staurosporine, which represented a positive control, decreased the viability of N2a cells to 49.9%. On the other hand, GRA3_Wh6_ showed a significant decrease in the number of viable N2a cells (*P* < 0.0001, 63.8% vs 80.7%) compared with the pEGFP-transfected cells (Fig. 3a). To further determine the rate of apoptosis, flow cytometry analysis using Annexin V-PE/7-AAD staining assay was performed. The results showed that GRA3_Wh6_-transfected N2a cells significantly promoted apoptosis (*P* < 0.0001, 44.8% vs 6.1%) compared with pEGFP-transfected N2a cells. The apoptosis rate in the mock-transfected cells was 5.2%, which was similar to that in pEGFP-transfected N2a cells (Fig. 3b).

### ER stress-mediated apoptosis induced by GRA3_Wh6_ in N2a cells

**Fig. 4:**
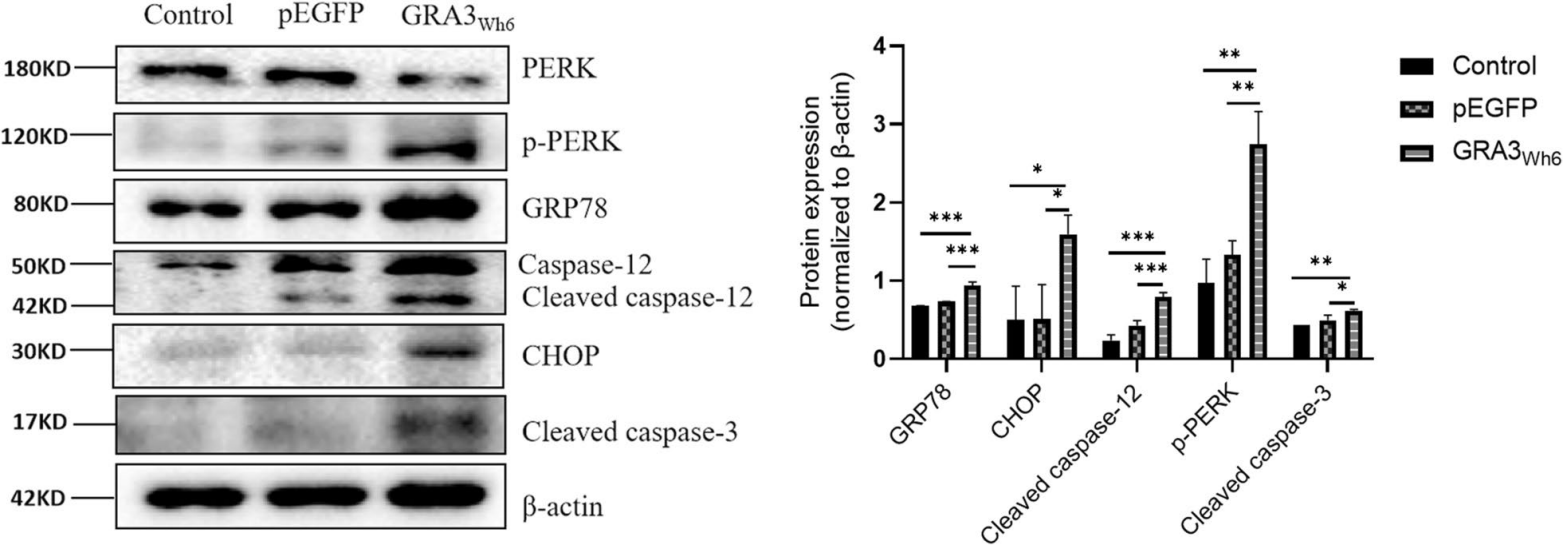
Expression of apoptosis-associated proteins and Endoplasmic reticulum stress (ERS) proteins induced by GRA3_wh6_ N2a cells were transfected with either pEGFP or pEGFP-GRA3_Wh6_ (GRA3_Wh6_) for a 24 h period. The expression levels of ER stress- and apoptosis-associated proteins were then determined by immunoblotting. Mock-transfected cells served as the negative control. The represented values were normalized and expressed relative to β-actin levels. CHOP C/EBP homologous protein, GRP78 78-kDa glucose-regulated protein, PERK PKR-like ER kinase, P-PERK phosphorylated PERK. Data were expressed as mean ± SD on three different assays (*n* = 3). *P < 0.05, **P < 0.01, ***P < 0.001

Our previous studies have identified certain effector proteins that can induce programmed cell death in mouse N2a and human choriocarcinoma JEG-3 cells via ER stress signaling pathways [10, 11, 20]. Here, we sought to determine whether the dense granule effector protein GRA3 activated ER stress-induced cell death. N2a cells were transfected with either pEGFP or pEGFP-GRA3_Wh6_ (GRA3_Wh6_), and the expression levels of ER stress-related proteins and apoptosis-associated proteins were assessed. Immunoblotting showed significantly increased ER stress-associated proteins, such as GRP78, an ER-associated molecular chaperone (*P* = 0.0002), and p-PERK (*P* = 0.0036) in GRA3_Wh6_-transfected cells compared with pEGFP-transfected cells. Consistent with the results obtained from ER stress-associated proteins, cells transfected with GRA3_Wh6_ showed elevated levels of apoptosis-mediated proteins such as C/EBP homologous protein (CHOP) (*P* = 0.0403), cleaved caspase-12 (*P* = 0.0010), and cleaved caspase-3 (*P* = 0.0196) compared with N2a cells transfected with pEGFP (Fig. 4). These results suggest that GRA3 of the Wh6 strain could induce ER stress-mediated apoptosis in neural cells.

### *Toxoplasma *GRA3 elevated the expression of ER stress- and apoptosis-associated genes

**Fig. 5:**
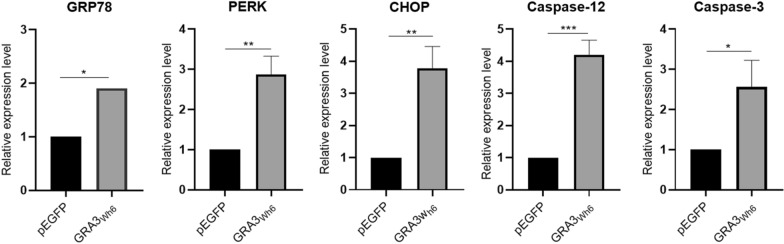
Transcription levels of apoptosis-associated genes induced by GRA3_wh6_. N2a cells were transfected with either pEGFP or pEGFP-GRA3_Wh6_ (GRA3_Wh6_) plasmid for a 24 h period. mRNA expressions of the associated apoptosis and ER stress genes were measured using RT-qPCR. The represented values were normalized and expressed relative to β-actin levels. GRP78 78-kDa glucose-regulated protein; CHOP, C/EBP homologous protein, PERK PKR-like ER kinase. Data were expressed as mean ± SD on three different assays (*n* = 3). *P < 0.05, **P < 0.01, ***P < 0.001

Following immunoblotting, we performed RT-qPCR to further compare the mRNA expression levels of the associated apoptosis and ER stress genes between pEGFP or pEGFP-GRA3_Wh6_ (GRA3_Wh6_)-transfected N2a cells. The results showed that GRA3_Wh6_-transfected cells had elevated mRNA levels of GRP78 (*t*_(2)_ = 2.800, *P* = 0.0488), PERK (*t*_(2)_ = 7.186, *P* = 0.0020), CHOP (*t*_(2)_ = 7.052, *P* = 0.0021), caspase-12 (*t*_(2)_ = 21.090, *P* = 0.0003) and caspase-3 (*t*_(2)_ = 4.066, *P* = 0.0153) compared with N2a cells transfected with pEGFP (Fig. 5).

### Attenuation of GRA3-induced N2a cell apoptosis following pretreatment with GSK2656157 and Z-ATAD-FMK

**Fig. 6:**
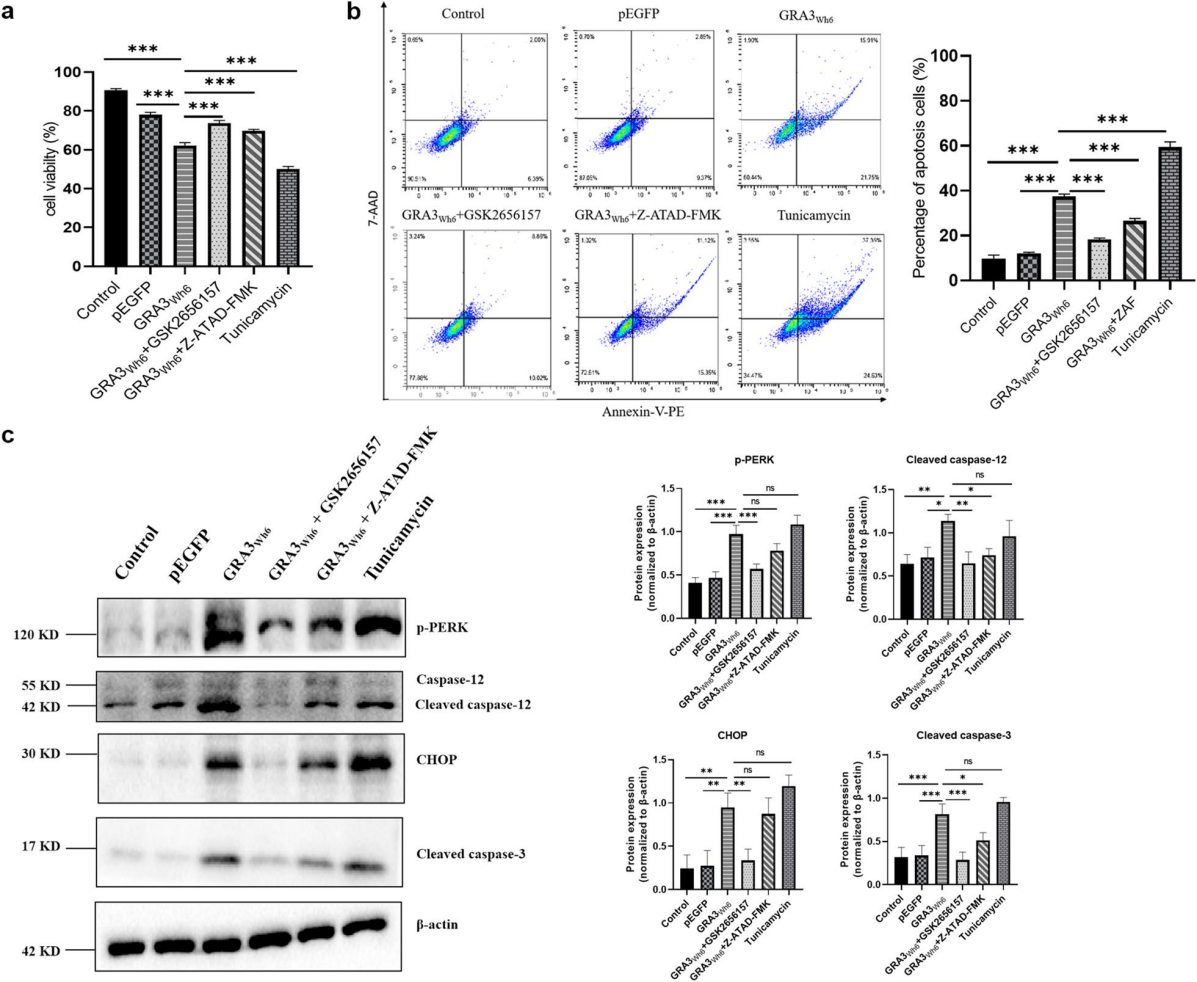
Effects of PERK and caspase-12 inhibitors on loss of cell viability and apoptosis in pEGFP-GRA3_wh6_ transfected N2a cells. N2a cells were treated with or without GSK2656157 (4 μM) and Z-ATAD-FMK (ZAF, 5 μM) for 1.5 h and 6 h, respectively, and transfected with either pEGFP or pEGFP-GRA3_Wh6_ (GRA3_Wh6_) for 24 h. **a** Cell viability was measured using the trypan blue staining cell viability assay. **b** Apoptosis of cells was determined using flow cytometry after staining with Annexin V-PE/7-AAD. The plots are from a representative measurement and the data were expressed as mean ± SD on three different assays (*n* = 3). **c** The protein expression levels of ER stress- and apoptosis-related proteins were determined by immunoblotting. The represented values were normalized and expressed relative to β-actin levels. The data were expressed as mean ± SD on three different assays (*n* = 3). *P < 0.05, **P < 0.01, ***P < 0.001, and ns, not statistically significant. GRA3_Wh6_ + GSK2656157 represents N2a cells pretreated with GSK2656157, followed by transfection with GRA3_Wh6_ plasmid. GRA3_Wh6_ + ZAF represents N2a cells pretreated with ZAF, followed by transfection with GRA3_Wh6_ plasmid

Cell viability and apoptosis were analyzed in GRA3_Wh6_-transfected N2a cells pretreated with GSK2656157 (PERK inhibitor, 4 μM, 1.5 h) and Z-ATAD-FMK (caspase-12 inhibitor, 5 μM, 6 h). Mock-transfected N2a cells showed 90.6% viability and an 8.4% apoptosis rate. pEGFP-transfected cells showed 78.0% viability and 12.3% apoptosis. Treatment with tunicamycin decreased N2a cell viability to 50.1% and increased the apoptosis rate to 61%. N2a cells transfected with GRA3_Wh6_ significantly reduced cell viability (*P* < 0.0001, 62.3% vs 78.0%) and increased cell apoptosis (*P* < 0.0001, 37.7% vs 12.3%) when compared with N2a cells transfected with pEGFP. Our results showed that pretreatment with GSK2656157 and Z-ATAD-FMK significantly increased cell viability (*P* < 0.0001 and *P* < 0.0001) and significantly decreased apoptosis (*P* < 0.001 and *P* < 0.0001) in GRA3_Wh6_-transfected cells (Fig. 6a, b). Furthermore, we analyzed the related proteins expressions of ER stress-associated apoptosis in GRA3_Wh6_-transfected cells following pretreatment with GSK2656157 and Z-ATAD-FMK. Immunoblotting results revealed that GRA3 increased the expression of cleaved caspase-12 (*t*_(2)_ = 5.236, *P* = 0.0110), cleaved caspase-3 (*P* = 0.0008), p-PERK (*P* < 0.0001) and CHOP (*P* = 0.0024) when compared with cells transfected with pEGFP. Compared with GRA3_Wh6_-transfected cells, the expression levels of p-PERK (*P* = 0.0008), cleaved caspase-12 (*P* = 0.0034), cleaved caspase-3 (*P* = 0.0003) and CHOP (*P* = 0.0051) were significantly decreased in GRA3_Wh6_-transfected cells pretreated with GSK2656157. Similarly, the protein expression levels of cleaved caspase-12 (*P* = 0.0169) and cleaved caspase-3 (*P* = 0.0284) were significantly decreased in Z-ATAD-FMK-pretreated GRA3_Wh6_-transfected N2a cells (Fig. 6c). Collectively, these results demonstrate that GRA3_Wh6_ induces ER stress-associated apoptosis via the PERK pathway.

## Discussion

*Toxoplasmosis* is known to be one of the most common parasitic infections that infect warm-blooded animals, including humans. Nearly a third of the human population have been chronically infected with *T. gondii* [25]. *T. gondii* can infect neural cells, where it forms cysts that remain for a lifetime in the host, causing chronic subclinical neuroinflammation [3, 26].

During cell invasion, secretory proteins such as dense granule proteins and rhoptry proteins (ROPs) are released into the host nucleus and cause considerable harm [27]. Several studies have explored how parasite proteins such as ROP16, ROP18, GRA15, and GRA60 contribute to *T. gondii* virulence either by activating or subverting host defense mechanisms [10, 20, 28]. Craver and Knoll sought to investigate the importance of GRA3 in the virulence of *T. gondii* [23]. To achieve this, they created a GRA3-deficient type II parasite to begin a functional characterization of the GRA3 protein. The GRA3 locus was disrupted by homologous replacement with a chloramphenicol resistance gene in a type II strain. Both in vitro and in vivo tests were performed to assess parasite growth and bradyzoite development and to assess the function of *T. gondii* GRA3 during infection. The result revealed no differences in the cell culture growth rate or bradyzoite formation between wild-type and ∆GRA3. Moreover, this study showed that the GRA3 monoclonal antibody derived after an RH infection does not react with the type II GRA3 protein because of a single amino acid change, indicating a difference in antigenicity between the two proteins, which is consistent with our result.

An important finding from the present study was that the GRA3 expression levels in avirulent type II ME49 and Chinese 1 Wh6 strains were significantly higher (*P* < 0.05 and *P* < 0.001, respectively) than those in the virulent type I RH and Chinese I Wh3 strains (Fig. 1). This was consistent with our previous study, which revealed that the expression level of GRA3 in the Wh6 strain was significantly higher than that in the RH and Wh3 virulent strains [29, 30].

The ER is responsible for the production of cellular organic molecules, including proteins, sterols, carbohydrates, and lipids [31–34]. Its role in protein folding is critical for cell survival. Cellular disturbances such as infections and reactive oxygen species can interfere with the normal functions of the ER. These cellular disturbances cause ER stress [35]. To alleviate this stress, the ER-localized transmembrane signaling proteins, i.e., inositol-requiring protein 1 (IRE1)-α, PERK, and activating transcription factor 6 (ATF6), activate UPR to restore cellular homeostasis [14, 15]. However, an unremitting UPR can activate UPR-mediated inflammatory and apoptotic pathways, resulting in cell death [16, 17]. We, for the first time in this study, demonstrate that the ER-*Toxoplasma* GRA3 interaction activates downstream apoptotic cascades in *T. gondii*-infected mouse N2a cells via the ER stress pathway. Therefore, our results indicate that GRA3 triggers neuronal apoptosis, which is not beneficial to the survival and dissemination of the parasites. The high expression of GRA3 in the less virulent strain might contribute to its lower virulence. Consistently, the low expression of GRA3 in the highly virulent strain helps it avoid neuronal apoptosis as much as possible, which helps its parasitism and widespread dissemination and may be one of the reasons for its strong virulence.

The protein folding function of the ER requires the presence of Ca^2+^-dependent ER molecular chaperone proteins. GRP78, commonly known as Bip, is one of the most-studied ER chaperone proteins [36]. Aside from being critical for protein quality control and thus sensing and targeting misfolded and/or unfolded proteins for degradation, GRP78 controls the activation of ER-stress transducers and acts as an ER stress sensor [37]. Within the cell, GRP78 levels are kept relatively low; however, they are upregulated in response to stressors that alter ER and Ca^2+^ homeostasis [38]. In our study, we demonstrated that *T. gondii* GRA3_Wh6_ induced ER stress in N2a, which significantly increased GRP78 mRNA and protein levels after a 24 h transfection with GRA3_Wh6_ (Figs. 4, 5). This observation is similar to our previous study, which involved the transfection of carcinoma JEG-3 cells with *Toxoplasma* GRA15_II_. In that study, pEGFP-GRA15_II_ increased the expression levels of GRP78. This suggests that the ER-GRA3 interaction induces ER stress, which in turn upregulates GRP78 expression levels in an attempt to restore homeostasis.

Caspase-12 plays a crucial role in ER stress-mediated cell death. Under ER stress conditions, pro-caspase-12 is cleaved, and the activated forms accumulate (Nakagawa et al*.*). Here, GRA3_Wh6_ was found to cleave pro-caspase-12 into active caspase-12, accelerating apoptosis. Having demonstrated that GRA3_Wh6_-induced ER stress activates caspase-12, we next examined the downstream targets of GRA3_Wh6_-induced apoptosis following caspase-12 activation. We observed that the activation of caspase-12 resulted in the activation of caspase-3, as demonstrated by the increased cleaved caspase-3 expression in GRA3_Wh6_-transfected mouse N2a cells. Our results showed that GRA3_Wh6_ induced the activation of caspase-12, which contributes to the pathogenesis of encephalitis during *T. gondii* infection*.* Moreover, pretreatment of N2a cells with the caspase-12 inhibitor Z-ATAD-FMK significantly decreased cleaved caspase-12 and cleaved caspase-3 protein expression levels; consequently, Z-ATAD-FMK downregulated apoptosis in GRA3_Wh6_ N2a cells.

As an important initiator of the unfolded protein response (UPR), PERK undergoes dimerization and autophosphorylation upon dissociation from GRP78. The kinase domain is then activated by phosphorylation of PERK, which then targets substrates such as eIF2a to activate the cascade [39]. The PERK signaling pathway is activated in response to excessive amounts of misfolded proteins in the ER and temporarily blocks protein translation, which results in neuronal cell death [40, 41]. Our results showed that GRA3_Wh6_ significantly activated ER stress and UPR, as observed by the significantly increased levels of phosphorylated PERK proteins following immunoblotting. Similarly, qPCR results showed that the mRNA levels of PERK were elevated in GRA3_Wh6_-infected N2a cells. Consistent with previous findings, our immunoblotting results showed that pretreatment of N2a cells with GSK2656157, a PERK inhibitor, significantly suppressed phosphorylated PERK expression. Trypan blue staining cell viability and Annexin V-PE/7-AAD apoptosis assays revealed that N2a cells that were pretreated with GSK26561157 also suppressed neuronal cell death 24 h after GRA3_Wh6_ transfection. Furthermore, inhibition of PERK downregulated CHOP, cleaved caspase-12, and cleaved caspase-3 expression. GSK2606414, a PERK inhibitor, was shown to have neuroprotective effects by rescuing the loss of dendritic development and number of synapses in neurons following traumatic brain injury and decreasing the expression of downstream targets such as phospho-eIF2a, ATF4, and CHOP [42, 43]. Therefore, initiation of UPR by a signal through the PERK pathway appears to play a crucial role in GRA3-mediated ER stress apoptosis.

CHOP plays a pathological role in ER stress-related diseases. During unremitting UPR, activation of PERK results in the phosphorylation of the eukaryotic translation initiation factor (eIF2), resulting in a general translational block. However, ATF4 (activating transcription factor 4) is translated, activating downstream targets such as C/EBP homologous protein (CHOP) [44]. PERK-ATF4-CHOP pathway activation during prolonged UPR induces apoptosis [45]. Our results showed that the mRNA and protein expression levels of CHOP in N2a cells were significantly increased 24 h after *T. gondii* GRA3_Wh6_ transfection, which translated into increased N2a cell death, as observed in cell viability and cell apoptosis flow cytometry assays. This further indicates that GRA3_Wh6_ induces ER stress and activates the PERK-ATF4-CHOP signaling pathway to induce apoptosis in neuronal cells.

## Conclusion

In conclusion, we have come a long way in our understanding of this protozoan parasite and its interaction with host cells. Our study highlights the mechanism by which dense granule protein (GRA3) increases the virulence of *T. gondii*. GRA3_Wh6_ induces neuronal apoptosis via the endoplasmic reticulum stress-mediated apoptosis pathway. This study provides further understanding of the mechanisms by which *T. gondii* causes neuropathology.

## Data Availability

Data are available from the corresponding authors upon reasonable request.

## Abbreviations

GRA3: Dense granule protein 3
CAML: Calcium modulating cyclophilin ligand
ER: Endoplasmic reticulum
UPR: Unfolded protein response
HIV: Human immunodeficiency virus
N2a: Neuro2a
ROP: Rhoptry protein
cDNA: Complementary DNA
mRNA: Messenger RNA
DNA: Deoxyribonucleic acid
RNA: Ribonucleic acid
GRP78: Glucose regulated protein 78
PERK: Protein kinase R (PKR)-like ER kinase
CHOP: C/EBP-homologous protein
SDS–PAGE: Sodium dodecyl sulfate–polyacrylamide gel electrophoresis
